# Development and implementation of explicit computerized protocols for mechanical ventilation in children

**DOI:** 10.1186/2110-5820-1-51

**Published:** 2011-12-21

**Authors:** Philippe Jouvet, Patrice Hernert, Marc Wysocki

**Affiliations:** 1 Pediatric Intensive Care Unit, Department of Pediatrics, University of Montreal, Montreal, Canada; 2 Research Center, Sainte-Justine Hospital, University of Montreal, Montreal, Canada

## Abstract

Mechanical ventilation can be perceived as a treatment with a very narrow therapeutic window, i.e., highly efficient but with considerable side effects if not used properly and in a timely manner. Protocols and guidelines have been designed to make mechanical ventilation safer and protective for the lung. However, variable effects and low compliance with use of written protocols have been reported repeatedly. Use of explicit computerized protocols for mechanical ventilation might very soon become a "must." Several closed loop systems are already on the market, and preliminary studies are showing promising results in providing patients with good quality ventilation and eventually weaning them faster from the ventilator. The present paper defines explicit computerized protocols for mechanical ventilation, describes how these protocols are designed, and reports the ones that are available on the market for children.

## Introduction

Mechanical ventilation is a sophisticated technique that can keep alive the most severely ill patients; however, it can simultaneously damage the lungs and unfortunately generate unwanted complications [1]. By analogy with pharmacology, mechanical ventilation can be viewed as a treatment with very narrow therapeutic windows, i.e., highly efficient but with considerable side effects if not used properly and in a timely manner. During the past two decades, considerable knowledge has been gained to find the optimal risk/benefit balance for mechanical ventilation. For instance, protective ventilation with low tidal volume (VT) and low airway pressure (Paw) has been shown to be safer than ventilation with high VT and Paw in adults with ARDS [2]. However, several publications in adults and children have reported variable effects of written protocols in implementing protective ventilation with relatively low compliance with use of the protocols [3,4] with a significant number of patients still being ventilated with high VT and high Paw [5,6]. Human and organizational factors are at least partially responsible for such poor compliance [7] but so are the vast diversity of patient types, conditions, and changes over time, which makes one protocol unable to fit all situations.

In addition, expertise and human resources are not always available to make sure that patients receive the best ventilation everywhere and all the time. The ability to make timely adjustments of the ventilator according to the patient's condition, without much inter-caregiver variability, would certainly improve the safety and efficiency of mechanical ventilation especially when resources and expertise are not at the bedside 24 hours per day. In the present paper, we will define an explicit computerized protocol, describe how these protocols are designed and developed currently, report the ones that are available on the market for children, and propose some considerations for future developments in this field.

## Definitions

*A protocol *is a document that is designed to guide decisions regarding diagnosis, management, and treatment of specific medical situations. The protocol is based on the medical knowledge acquired from physiological studies, expertise, or evidence and can be generated by individuals or by consensus obtained from a group of physicians or experts. Protocols often are not precise enough to generate a decision at the bedside in a specific situation and thus significant inter-clinician variability in their application may exist.

*An explicit protocol *is designed to provide enough details to generate patient-specific therapy instructions that can be performed by different clinicians with no inter-clinician variability [8]. Important individualization of patient therapy can be preserved by explicit protocols when they are driven by individual patient data. Considering the number of clinical situations and inputs from a given patient, explicit protocols rapidly become so complex that computers are required to integrate the large amount of information and provide specific answers to the user.

*An explicit computerized protocol *(ECP) is an explicit protocol supported by computer science to apply the instruction for a given patient at a given time. ECP might be in a laptop or integrated into the ventilator or monitoring device. The medical knowledge is usually implemented in the ECP through "*if... then*" rules. For example: *if *the SpO_2 _is < 88%, *then *increase FiO_2 _by 10%. The rules can be more complex and based on a validated physiological equation, such as the Otis equation [9] as used in IntelliVent™ (Hamilton, Bonaduz, Switzerland).

A rule can be in an open or closed loop. The rule is an open-loop rule if it results in a therapeutic or diagnostic recommendation displayed on the screen of a device for which caregivers can agree whether to accept the recommendation. According to the previous definitions, a *clinical decision support system *(CDSS) is defined as an ECP that uses two or more items of patient data to generate case-specific recommendations through rules that are only in open-loop [10].

**Figure 1:**
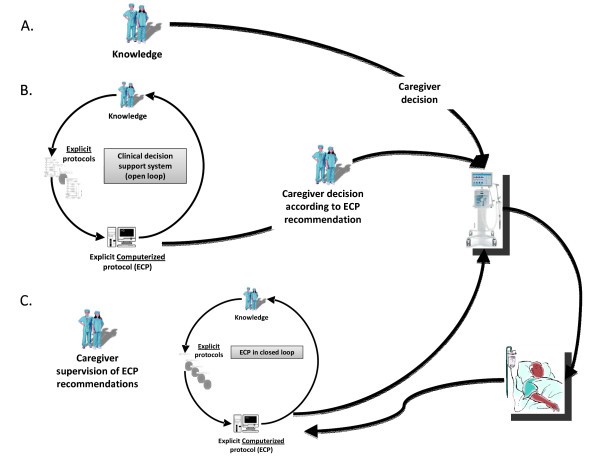
**Schematic representation of the different processes for decision making**. **(A) **Actual caregiver decision making. **(B) **Explicit computerized protocol in open-loop (clinical decision support systems). **(C) **Explicit computerized protocols in closed-loop.

As a step further, a rule that provides a recommendation of modification of the ventilator setting and implements this modification without caregiver intervention is a rule in closed-loop. Currently most of the ECPs used commercially for mechanical ventilation involve both open and closed-loop ventilation rules. A closed-loop ECP is arbitrarily designated as an ECP with at least one rule in closed-loop (Figure 1).

## Why do we need explicit computerized protocols for mechanical ventilation?

The human brain has a limited ability to incorporate data and information in decision making and human memory can simultaneously retain and optimally utilize only seven plus or minus two data constructs [11]. The amount of data that is retained is even less when caregivers are working at night, with stress and/or time pressure. This limitation contrasts sharply with the clinical reality in which hundreds of variables are encountered by the caregivers in the ICU setting and decisions are made 24 hours per day. To prescribe mechanical ventilation, numerous parameters are considered, including all physiological data provided by the respirator, monitors, and clinical data from the charts on diagnosis and use of sedatives and hemodynamic treatments. The mismatch between human ability and the vast amount of data and information contributes to variation in clinical practice as decisions are made applying different data constructs and different knowledge/expertise. In such a complex environment, the help of an ECP is crucial to limit inter-caregiver variability. In a less complex environment, the aviation industry confronted human factors responsible for accidents [12] and decided decades ago to develop and implement closed-loop ECP in airplanes to improve safety resulting in a model of safety management today. That being said, in the medical field, the major limitation to developing ECP is to agree on which medical knowledge to implement.

## Development of explicit computerized protocols

A multidisciplinary approach is needed to generate an ECP; the team should include clinical expert(s) in mechanical ventilation to generate the knowledge and validate ECP in a clinical environment, computer scientists to design the ECP platform, biomedical engineers to implement the ECP into medical devices (monitors and/or ventilators) and test ECP robustness and reliability, and industry to finalize a product that will receive a European Community marking (CE mark) and a U.S. marking (Food and Drug Administration (FDA)) approval.

### Generation and validation of medical knowledge implemented in an ECP

The basic component of an ECP is a medical knowledge-based rule. In our clinical practice, we continuously apply rules. If we take the previous example of the SpO_2_/FiO_2 _rule, caregivers modify FiO_2 _according to SpO_2 _routinely. An ECP will recommend (open-loop) or do (closed-loop) in the same way, as soon as a valid SpO_2 _is available. The ECP also will define how often the FiO_2 _can be changed, the amplitude of change, and add additional rules: for example, define what will occur if FiO_2 _is 100% and SpO_2 _still below normal range.

The knowledge needed to develop an ECP able to manage the course of mechanical ventilation in any ICU patient is vast and we all know that "the devil is in the details." This knowledge is based on published work on respiratory physiology, clinical observational studies to describe current practice [6,13], consensus conferences to define the specific clinical decision points (for example when do pediatric intensivists consider that we should switch from conventional mode to high-frequency ventilation mode in ARDS patients?) and clinical trials to validate ECPs [4,14,15]. A step-by-step approach, including more than one research center, is needed to develop valid, robust, and widely accepted ECPs. For example, the medical knowledge acquired in the past two decades on weaning in pressure support mode resulted in the development of SmartCare/PS™. SmartCare/PS™ was developed by one research team, and more than a decade elapsed from conception to commercialization [16,17]. ECPs based on SpO_2_/FiO_2 _have already been developed for neonates and children and need further clinical validation [14,15].

To shorten development and validation times, one option would be to follow the same development and validation process used in aviation by using a simulated flight environment (wind, temperature...). Medical ECP would need virtual patients with realistic physiological and pathological behaviors for developing and validating ECPs before clinical trials. Several teams are already working on such platforms, although none are currently commercialized for this purpose [18-20].

### ECP platform

**Figure 2:**
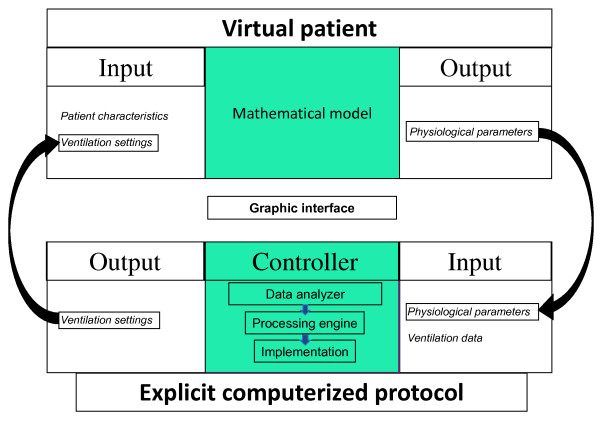
**The five components of a platform for development of an explicit computerized protocol (input data, controller, output data, graphic interface, virtual patient)**. The explicit computerized platform collects the data from the patient (SpO_2_, ET_PCO2_, ventilation data...) and processes the data to determine new ventilator settings in open- or closed-loop (output data). The virtual patient simulates the breathing pattern and the resulting blood gases for a mechanically ventilated patient with predefined characteristics (age, body weight, lung compliance, cardiac output, ventilator settings...). This virtual patient helps to test a large panel of clinical situations to validate the rules implemented and to detect any bugs.

The five technical components (Figure 2) of an ECP are: 1) input data (entered manually or captured electronically from devices); 2) a control unit that analyses the input data to generate orders; 3) output data; 4) an interface that display a recommendation (open-loop) or implements the setting modification (closed-loop); and 5) a virtual patient as mentioned above.

In the SpO_2_/FiO_2 _rule already described, the *input data *is patient SpO_2_, the control unit analyses SpO_2 _and selects the rule that corresponds to SpO_2 _value (e.g., increase FiO_2 _if SpO_2 _is low, decrease FiO_2 _if SpO_2 _is high (knowing that oxyhemoglobin dissociation curve is flat at SpO_2 _> 97%), or no change if SpO_2 _is in normal range), the output data is the FiO_2 _suggestion displayed on a screen (open-loop) or a setting modification on the respirator (closed-loop).

The input data, whether entered manually by the clinician or captured by a medical device, needs to be processed to discard artefacts and to be clinically relevant before being sent to the control unit [21]. The key point is to input relevant, valid, robust, and stable data for the ECP. For example, the weaning ECP for children (SmartCare/PS™, Dräger, Germany) transforms real-time tidal volume, respiratory rate, and end tidal PCO_2 _(ET_PCO2_) into mean values during a 2-minute period. With IntelliVent™ (Hamilton Medical, Switzerland), the ET_PCO2 _used for the minute ventilation closed-loop is the second highest breath-by-breath ET_PCO2 _with enough quality index during the last 10 breaths [14]. On the other hand, ET_PCO2 _is not always a good surrogate for PaCO_2 _especially during the acute phase of illness with high dead space. Such situations must be detected by the ECP to avoid any misinterpretation of the input data.

The control unit receives clinical information from the patient (input data) and "transforms" this information into orders (output data). The basic structure for processing information is rule-based (see above). All potential paths and situations should be addressed to lead to specific instructions. It usually requires several set of rules to manage two to three input data each. For example, a first set of rules could be for pressure support management according to tidal volume, respiratory rate, and ET_PCO2 _[17], a second set of rules could be for FiO_2 _and PEEP management according to SpO_2 _[22], and another set of rules could be for recommending extubation when pressure support, PEEP, and FiO_2 _reach threshold values for a certain length of time (mimicking an extubation readiness test) [4]. Rules are usually "*if... then *..." rules, but some ECPs have been developed using fuzzy logic [23], i.e., integrating patient's information in a fuzzy way to mimic the human brain [24,25]. Despite the use of fuzzy logic in aircraft autopilot and in various other applications [25], to the best of our knowledge, fuzzy logic is not used in commercialized medical ECPs.

Output data can be recommendations to the caregivers suggesting new ventilator settings, specific orders such as "patient ready for separation from the ventilator," or ventilator settings being automatically adjusted. During the development phase of an ECP, output data are usually recommendations (open-loop). After extensive testing, some rules or sets of rules can be switched to close the loop.

A simple, attractively presented, and intuitive user interface is crucial to facilitate the understanding of the ECP decision process and for knowledge transfer at the bedside [26]. Ideally, the user interface should include educational tools to train caregivers on mechanical ventilation management according to the ECP, as done with simulators for aircraft pilots.

As mentioned above, use of a virtual patient mimicking patient-ventilator cardiorespiratory interactions also is important for the development of ECPs. As for aircraft autopilot, a virtual patient may help "debug" the very first ECP versions but also aid understanding the complex interactions between rules and achieving preclinical validation. In addition such virtual patients, which should ideally be incorporated in the medical device (i.e., the ventilator), might be more efficient in training and teaching the eventual users (Figure 2). Currently, the virtual patients used for ECP development are computer simulations of the physiologic processes of respiration and circulation, using mathematical models.

Several barriers exist to the development of ECPs: 1) We do not generate enough medical knowledge in mechanical ventilation and most of the time ECPs are targeting a relatively small and regional scientific community. This can be improved by promoting multicenter international collaborations along the lines of the Pediatric Acute Lung Injury and Sepsis Investigators Network (PALISI), the European Society of Pediatric Intensive Care Medicine and Collaborative Critical Care Research Network (CCCRN) [5,27]; 2) It is important to be able to capture any refusal of an ECP recommendation and to analyse if an adjustment of the ECP is mandatory. The commercialized ECP described below do not have such reporting systems. In the future, the ECPs should be equipped with a data report system and an expert team should analyze the data to refine the ECP. The versatility and the ease in upgrading and adjusting the ECP are probably key factors in making ECP widely accepted; 3) The manufacturers are unfortunately not ready to share their ECPs, processes and knowledge, for obvious marketing and business reasons. They also may need more resources and less time-to-market constraints to innovate further. Consortium(s) like those that exist in aeronautics could be of considerable value in driving forward innovation in this field.

Several barriers also exist to the acceptance and implementation of ECPs. These barriers include the lack of awareness, lack of familiarity with the protocol, lack of agreement, lack of demonstrated safety and efficacy, lack of known improved outcome, lack of ability to overcome the inertia of previous practice, protocol-related barriers (not easy to use, not convenient, cumbersome, confusing), environment-related barriers (new resources or facilities not accessible) [8]. Among all these barriers, the safety issue is the first and most important one. The safety issue is addressed using the three following principles: 1) ECPs suggests a modification or modifies ventilation settings only within the alarms prescribed on the respirator; 2) there are stop rules implemented in ECPs that interrupt closed loop protocols in specific situations (if input data are not available for example); 3) Most of the ECPs in closed loop are first developed and tested for the management of the weaning phase. Despite these barriers many set of rules even in closed loop are widely accepted by caregivers. For example, the algorithm of the neurally adjusted ventilatory assist mode (NAVA) includes a rule that automatically adjusts positive inspiratory pressure to patient's electrical activity of the diaphragm change to deliver ventilation proportional to patient's needs.

## Explicit computerized protocols for mechanical ventilation in children

Saxton and Myers reported the first ECP to adjust the end tidal PCO_2 _by regulating the negative pressure of an iron lung ventilator in poliomyelitis patients [28]. Pediatricians then soon became concerned with adjusting the FiO_2 _tightly in order to avoid hypo/hyperoxemia and their related side effects [29,30]. Dugdale and coworkers [31] reported in 1988 seven neonates with respiratory distress syndrome treated during 48 hours with a closed-loop FiO_2 _adjustment to keep the PaO_2 _obtained from an indwelling umbilical artery electrode at 10 kPa. The time spent by the neonates with PaO_2 _at ± 1 kPa of the target PaO_2 _was 75% with closed-loop control FiO_2 _compared with 45% with manual adjustment of FiO_2_. But the invasiveness of the PaO_2 _monitoring precluded the development of this ECP. Claure et al. conducted a research program designed to develop closed-loop FiO_2 _adjustment using SpO_2 _[32,33]. These authors recently conducted a multicenter, randomized, clinical trial that included mechanically ventilated preterm infants who were ventilated during two consecutive 24-hour periods: one with FiO_2 _adjusted by caregivers and the other by an automated system, in random sequence. Automated FiO_2 _adjustment improved maintenance of the intended SpO_2 _range, and led, significantly, to reduced time with high SpO_2 _and more frequent episodes with SpO_2 _between 80% and 86% [15]. This ECP is now commercialized as CLiO2™ (CareFusion, Yorba Linda, US) and implemented in a respirator.

SmartCare/PS™ (Draeger Medical, Lübeck, Germany; PS stands for pressure support) is an ECP for the closed-loop control of pressure support ventilation. This ECP operates without the need of caregivers intervention but under their supervision (Figure 1), the four following therapeutic procedures: 1) automatic adaptation of the pressure support level to keep the patient inside a "zone of respiratory comfort" that corresponds to a respiratory pattern that is determined by lower and upper thresholds of tidal volume, respiratory rate, and end tidal PCO_2_. These thresholds are defined within acceptable limits as established by a large panel of pediatric intensivists [34]; 2) a strategy to gradually and progressively decrease the level of pressure support level; 3) an automated spontaneous breathing trial (SBT) when the patient reaches a minimum ventilation support; and 4) a recommendation of separation from the ventilator when the SBT is successfully passed (Table 1). SmartCare/PS™ is available on Draeger's Evita XL and the latest generation of Draeger Medical ventilators: Evita Infinity V500. Among the first 20 pediatric patients treated with SmartCare/PS™, median time in "zone of respiratory comfort" was 91% (range, 0.7-99%) [4]. In a single-center, randomized, clinical trial (RCT), a significant decrease in weaning duration in the SmartCare/PS™ group (n = 15) was observed compared with usual care (n = 15), without any modification in weaning failure rates [35]. A multicenter RCT should be conducted because the benefit of SmartCare/PS™ may vary from one PICU to another as suggested by studies done in adults [16,36]. The major strength of SmartCare/PS™ is the implementation of an ECP for mechanical ventilation with a user-friendly interface that allows individual customization. There are several improvements to consider for SmartCare/PS™: 1) children with IBW < 15 kg are excluded. Therefore, another ECP is needed in the same PICU for infants < 15 kg. 2) PEEP and FiO_2 _are not automatically adjusted by SmartCare/PS™ but are part of the recommended criteria to initiate a spontaneous breathing trial (SBT).

**Table 1:** Characteristics of the SmartCare/PS^™ ^Draeger Medical and IntelliVent^™ ^Hamilton Medical explicit computerized protocols

Characteristics	SmartCare/PS™	IntelliVent™
Ventilation mode	PSV	ASV
Type of breath	PS	PC and PS
Body weight range for use	≥15 kg	≥7 kg
Primary goal of the ECP	wean while maintaining ET_PCO2_, RR, Vt within predefined range	maintaining ET_PCO2_, RR, Vt, SpO_2_ within predefined ranges
Initial settings	IBW, humidification system, medical history	IBW, medical history
Clinical decision support Option (open-loop)	No	Yes
Input data	_2 min_ET_PCO2_, _2 min_RR, _2 min_Vt, PEEP, PS level,	ET_PCO2_, RR, Vt, SpO_2 _PEEP, PIP, breath by breath
Output data	PS level,	MV, PEEP, FiO_2_
SBT	Yes	Yes
Recommendation for separation from the ventilator	Yes	SBT duration displayed

Adapted from Jouvet et al. [39]

PS, pressure support; PC, pressure controlled; ASV, adaptive support ventilation; IBW, ideal body weight; Vt, tidal volume; RR, respiratory rate; ET_PCO2_, end tidal PCO_2_; SpO_2_, pulse oxygen saturation; _2 min_Vt, _2 min_RR, or _2 min_ET_PCO2_, mean value on 2 min of Vt, RR, or ET_PCO2_; MV, minute volume; PEEP, positive end expiratory pressure; FiO_2_, inspired fraction of oxygen; PIP, positive inspiratory pressure; SBT, spontaneous breathing trial.

IntelliVent™ (Hamilton Medical, Bonaduz, Switzerland) is an ECP for the automated control of minute volume, PEEP, and FiO_2 _in adaptive support ventilation (ASV). IntelliVent™ manages, with (open-loop) or without the need of caregivers intervention (closed-loop), the four following weaning steps: 1) switch from control ventilation to spontaneous breathing (specific to ASV mode); 2) automatic adaptation of the pressure to maintain the patient in a range of respiratory rate, end tidal PCO_2 _and SpO_2_; 3) an automated SBT when children reach minimum ventilation support; and 4) a timer that shows SBT duration (Table 1). IntelliVent™ is available on the G5 and S1 generation ventilators from Hamilton Medical that continuously monitor usual mechanical ventilation parameters plus ET_PCO2 _and SpO_2 _[14]. IntelliVent™ has been assessed in one clinical trial on feasibility and safety in children during the weaning phase. Fifteen children were included and IntelliVent™ was safe and kept patients with body weight ≥7 kg in the "zone of respiratory comfort" comparably to PSV or ASV [14]. The major strength of IntelliVent™ is the combination of an ECP and user-friendly interface that allows a certain individual customization of the protocols, including automatic recruitment maneuvers. There are several improvements to consider for IntelliVent™ in children: 1) at present, there is not much clinical experience with ASV and this ECP in children; 2) children with ideal body weight < 7 kg are excluded, therefore, another ECP is needed in the same PICU for younger children; 3) automatic PEEP adjustment needs additional validation; 4) IntelliVent limits adjustments of PEEP in the case of hemodynamic instability based on the presence of pulsus paradoxus on pulse oximetry waveform (heart lung index) [37,38]. The accuracy of the waveform assessment and the upper limits of PEEP adjustment need to be validated in children.

### New ECP developments

**Figure 3:**
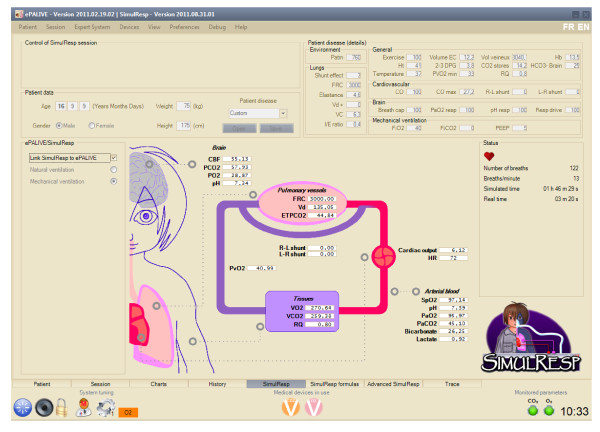
**Example of a platform for development of an explicit computerized protocol dedicated to the management of mechanical ventilation in children with acute lung injury that includes a virtual patient (named SimulResp) connected to a platform where rules are implemented (personal data)**.

A research program designed to develop and validate an ECP for the management of mechanical ventilation in children with acute lung injury is being conducted by a working group of the PALISI network in collaboration with CCCRN. This program is based on the following principles: the ECP will be an adaptation of a protocol already developed in adults (ARDS Network); the applicable type of ventilation will be pressure control mode and high frequency oscillatory (HFOV) mode. The computerized protocol will start at initiation of ventilation. The basic structure of the protocol will be that of a closed-loop system with the initial stage of design being open-loop, even for HFOV. The input data will include ventilator data, ET_PCO2_, SpO_2_, blood gases, and an automatic analysis of chest x-ray. The output data will be ventilation settings modifications and specific recommendations. Several preliminary studies are ongoing to refine the input data [13]. The ECP will be first developed on a laptop. To retain simplicity, the design will be closed-loop, so the final product will require few interventions from caregivers. A data report system will register the reason why the decision was not approved during the open-loop phase and should register other data, including accurate time and version of the protocol being used. Downloading the data should be possible in a database format and iterative refinement will be performed by a consensus committee. Preliminary data on the ECP has already been collected [6] and an ECP platform is almost completed (Figure 3).

## Conclusions

Considering the current context in the PICU (manpower shortages, increased complexity of care, lack of specific knowledge, needs for quality, safety, and reproducibility), ECP for mechanical ventilation will very soon be a "must." Open-loop and decision support systems allow too much room for inter-physicians variability and may not achieve the goal of providing the best possible care. As in many other fields, safety and quality will be achieved by closing the loop but allowing physicians to retain the role of supervisor, most directly involved with the most severe or atypical patients. Several closed-loop systems are already on the market, and preliminary studies have shown promising results in providing patients with good quality ventilation and eventually weaning them faster from the ventilator.

## Competing interests

PJ has a research salary from the "Fonds de Recherche du Québec - Santé" in respiratory critical care. PJ's research on virtual patient (SimulResp) is funded by a grant from the "Natural Sciences and Engineering Research Council of Canada." The clinical research conducted by PJ on SmartCare™ was funded by the Research network of the "Fonds de Recherche du Québec - Santé" and a Ventilator S1 Evita 4 was provided for research purposes by Draeger Medical. The clinical research conducted by PJ on IntelliVent™ was funded by a grant from Hamilton Medical, and a respirator S1 was provided for research purposes by Hamilton Medical. PJ was invited twice to present the results of the clinical research on IntelliVent™ at international meetings organized by Hamilton Medical. PJ also had a respirator Servo i, provided for research purposes by Maquet Medical.

MW was the director of the Research and Development Department of Hamilton Medical. MW is cosharing a patent on IntelliVent™ (WO/2007/085110 and WO/2007/085108).

PH's salary came from grants from the Research network of the "Fonds de Recherche du Québec - Santé" and the "Natural Sciences and Engineering Research Council of Canada."

## Authors' contributions

PJ, PH, and MW drafted the manuscript. All authors read and approved the final manuscript.
